# Imaging-defined complete response to immune checkpoint inhibitors predicts durable survival in advanced hepatocellular carcinoma

**DOI:** 10.3389/fonc.2025.1687449

**Published:** 2026-02-11

**Authors:** Yongjie Shao, Junhui Yuan, Yanwei Liu, Feng Wang

**Affiliations:** 1 Department of Interventional Radiology, The First Affiliated Hospital of Dalian Medical University, Dalian, Liaoning, China; 2 Department of Interventional Radiology, The University of Hong Kong–Shenzhen Hospital, Shenzhen, Guangdong, China; 3 Department of Medical Imaging, The Affiliated Cancer Hospital of Zhengzhou University & Henan Cancer Hospital, Zhengzhou, Henan, China; 4 Department of Ultrasound, Southern University of Science and Technology Hospital, Shenzhen, Guangdong, China

**Keywords:** complete response, hepatocellular carcinoma, immune checkpoint inhibitors, mRECIST, survival outcomes

## Abstract

**Background:**

Immune checkpoint inhibitors (ICIs) have revolutionized advanced hepatocellular carcinoma (HCC) treatment, yet complete responses (CRs) remain rare, and their long-term outcomes are poorly characterized. This study evaluates clinical outcomes, pathologic correlates, and optimal management strategies for HCC patients achieving CR to ICI-based therapy.

**Methods:**

We retrospectively analyzed 160 patients with advanced HCC who attained CR (70 by mRECIST; 90 by RECIST v1.1) following ICI therapy at four tertiary centers. Outcomes included recurrence-free survival (RFS), overall survival (OS), and pathologic validation of radiographic CR. Multivariable Cox models identified predictors of RFS.

**Results:**

CRs occurred in 4.8% of treated patients. The cohort demonstrated exceptional survival, with 3-year OS and RFS rates of 86% and 55%, respectively. Among the 8 patients who underwent resection or liver transplantation, 6 (75%) achieved pathologic complete response, 2 in the CR-RECISTv1.1 group and 4 in the CR-mRECIST-only group, supporting imaging validity. Multivariable analysis revealed presence of macrovascular invasion (aHR 2.47, p=0.003) and presence of extrahepatic metastases (aHR 2.00, p=0.011) as independent risk factors for recurrence, while CR by RECIST v1.1 predicted improved RFS (aHR 0.62, p=0.015). Patients continuing ICI ≥6 months post-CR had superior 3-year RFS (81% vs. 55%, p=0.002). Of 11 patients undergoing curative conversion therapy (resection/transplantation/ablation), 92% survived at 3 years with 75% RFS.

**Conclusions:**

CR to ICI therapy, though uncommon, correlates with unprecedented survival in advanced HCC, even among high-risk subgroups. mRECIST-defined CR shows strong pathologic concordance, addressing concerns about anti-angiogenic confounders. Extended ICI duration post-CR and selective conversion therapy may optimize outcomes. These findings redefine prognostic paradigms and underscore the need for biomarker-driven strategies to sustain remission.

## Introduction

Hepatocellular carcinoma (HCC) is one of the most common and deadly cancers worldwide. HCC is often diagnosed at a localized late stage or metastatic stage, where systemic therapy is recommended if liver function is preserved (1). Immune checkpoint inhibitors (ICIs) have become a cornerstone of first-line systemic treatment for advanced HCC, representing the current standard of care (2). Although ICIs are not considered curative, they can induce profound and durable responses in some patients. This may enable sequential treatment stage migration, potentially allowing for curative conversion therapies—such as resection or liver transplantation—in select patients initially diagnosed with advanced HCC. A small subset of patients may even achieve a complete response (CR) with ICI-based combination therapy (3).

Despite the increasing use of immunotherapy in HCC, little is known about the long-term outcomes of patients who achieve a CR following ICI treatment (4). Currently, there is no established clinical guidance on how to manage these patients after CR, leaving several critical questions unresolved. Key uncertainties include whether it is safe to discontinue immunotherapy once CR is achieved—and if so, at what point—as well as whether patients should be considered for curative-intent conversion therapies (such as resection or transplantation) or instead maintained under close surveillance for recurrence.

Another area of debate is the optimal method for assessing CR in the context of immunotherapy. While mRECIST is widely used in HCC, its ability to reliably identify complete tumor necrosis following ICIs remains uncertain (5). Some argue that true CR should be defined strictly by RECIST v1.1 criteria, which require the complete disappearance of all target lesions (6–8). Clarifying these questions is crucial, as achieving CR may correlate with unprecedented long-term survival, and optimal post-CR management could further improve outcomes.

To address these knowledge gaps, we conducted a large-scale, retrospective, multicenter study. Our primary objective was to evaluate the clinical outcomes and optimal management strategies for this unique subset of HCC patients—those who attained CR after ICI-based systemic therapy.

## Methods

### Ethical approval

This study was approved by Dalian Medical University Institutional Research Committee, and written informed consent for medical research was obtained from all patients before starting the treatment. All methods were performed in accordance with the relevant guidelines and regulations.

### Patient selection

We conducted a retrospective, multicenter analysis of patients with advanced HCC who achieved a radiographic CR following ICI-based systemic therapy. The study included 160 patients (143 males [89%] and 17 females [11%]) treated between 2015 and 2025 across four tertiary medical centers in China (Henan Cancer Center, Southern University of Science and Technology Hospital, The First Affiliated Hospital of Dalian Medical University, and Shenzhen Qianhai Shekou Free Trade Zone Hospital). Eligible patients had radiologically or histologically confirmed HCC, received ICI therapy in a non-curative setting as determined by a multidisciplinary tumor board, and subsequently achieved a radiographic CR assessed by either mRECIST or RECIST v1.1 criteria. All enrolled patients were required to have viable tumor tissue present at the initiation of ICI therapy. We excluded patients who received ICI therapy in the (neo)adjuvant setting (before or after surgical resection or ablation) or those who underwent concurrent local therapy during systemic ICI treatment.

### Variable definition

We defined ‘therapeutic conversion therapy’ as any treatment modality administered to patients who achieved CR with the intent of inducing long-term remission. Pathological complete response (pCR) was rigorously defined as the absence of any viable tumor cells upon comprehensive histological examination of the entire resected or explanted liver specimen. All specimens were evaluated independently by two specialized hepatobiliary pathologists. Histological sections were assessed using hematoxylin and eosin (H&E) staining to identify viable tumor cells, which were characterized by nuclear hyperchromasia, prominent nucleoli, and a high nuclear-to-cytoplasmic ratio. The specimens were extensively sampled, with an emphasis on areas corresponding to the original tumor location on pre-treatment imaging. The predominant findings in cases achieving pCR were extensive necrosis and/or fibrotic scar tissue. In cases with discordant imaging and pathological findings, additional immunohistochemical staining for CD8+ T-cells and PD-L1 expression was performed to characterize the tumor immune microenvironment. A case was designated as a pCR only upon consensus agreement between both pathologists. Any surgical intervention performed following tumor recurrence was classified as treatment for relapse.

### Imaging assessment and follow-up

Standardized imaging follow-up was performed at predefined intervals for all patients across the participating centers. Contrast-enhanced CT or MRI was conducted at baseline, then every 6-9 weeks during the first year of treatment, and every 12 weeks thereafter until disease progression or treatment discontinuation. Both RECIST v1.1 and mRECIST criteria were applied to the same set of imaging studies at identical time points for every patient. This synchronous, dual-criteria assessment by central review ensured consistent and comparable evaluation of radiographic responses throughout the study period.

### Statistical analysis

We analyzed baseline characteristics using standard descriptive statistics. The median follow-up time was calculated through the reverse Kaplan-Meier method to account for censored observations. Treatment duration was determined as the interval between immunotherapy initiation and the final administration date, with patients continuing treatment at last follow-up being right-censored in our analysis.

For response assessment timelines, we established the date of first complete radiological response differently for each criterion: for the overall cohort and CR mRECIST subgroup, we used the initial imaging study demonstrating complete response by mRECIST standards (disappearance of all intratumoral arterial enhancement); for the CR-RECISTv1.1 subgroup, we applied RECIST v1.1 criteria (complete disappearance of all target lesions). We defined three key endpoints in our survival analysis: (1) Recurrence-free survival (RFS) measured from first radiological CR to either tumor recurrence or death, whichever occurred first, with living patients without recurrence censored at last contact; (2) Response duration calculated from first CR to tumor recurrence, censoring patients who died before recurrence at their date of death and those remaining recurrence-free at last contact; and (3) Overall survival (OS) determined from ICI initiation until death, censoring surviving patients at final follow-up.

We generated survival curves using the Kaplan-Meier method with between-group comparisons performed via log-rank testing. Our regression analysis included both univariate and multivariate Cox proportional hazards models, with the multivariable model constructed through stepwise backward elimination of non-significant covariates (retention threshold p<0.05).

To mitigate immortal time bias when assessing the impact of post-CR treatment duration, a landmark analysis was performed. A landmark time was set at 6 months after the date of first documented CR. Only patients who were alive and recurrence-free at this 6-month landmark were included in the analysis. These patients were then categorized based on their treatment status at the landmark: those who had received ICI therapy for ≥6 months after CR (and were typically still on treatment) versus those who had discontinued ICI before completing 6 months of post-CR therapy. RFS was compared between these two groups from the 6-month landmark time onward using the Kaplan-Meier method and log-rank test.

All statistical computations were performed using IBM SPSS Statistics (v26.0, SPSS Inc., Chicago, IL), R statistical software (v4.3.1, R Foundation for Statistical Computing, Vienna, Austria), and GraphPad Prism (v10.2.1, GraphPad Software, San Diego, CA) for graphical representations. We considered two-tailed p-values <0.05 statistically significant throughout all analyses.

## Results

### Patient characteristics and treatment patterns

Between September 2015 and November 2023, 3,333 patients received ICI therapy for advanced HCC in a non-curative setting. From this population, 160 (4.8%) achieved a complete response and comprised the study cohort, including 70 with a CR by mRECIST and 90 with a CR by RECIST v1.1. The population had a mean age of 65 ± 10 years, with predominantly male patients (n=143, 89%) and Child-Pugh A liver function (n=147, 92%). Disease staging revealed 70.6% of patients (n=113) had Barcelona Clinic Liver Cancer (BCLC) stage C disease, while 25.6% (n=41) were BCLC stage B. The four BCLC stage A patients (2.5%) received ICI-based therapy due to: patient preference (primarily for cases with recurrent disease after multiple local therapies), unfavorable tumor location for local treatment, or concerning tumor biology evidenced by high alpha-fetoprotein levels and paraneoplastic erythrocytosis.

Viral hepatitis represented the most common underlying liver disease etiology, with 56.3% (n=90) having HBV- or HCV-related disease (HBV: n=56; HCV: n=34). Among HBV patients, 86.2% (n=50) were on antiviral therapy before ICI initiation, while 6.9% (n=4) started antivirals during or after ICI treatment. The remaining 6.9% (n=4) had undetectable HBV DNA at baseline. For HCV patients, 22.2% (n=8) had detectable HCV RNA at baseline, with 8.3% (n=3) receiving antiviral therapy during or after ICI treatment, while 13.9% (n=5) remained untreated.

Prior to systemic therapy, 80% of patients (n=128) had undergone surgical or local-regional treatments (e.g., TACE: n=87; resection: n=64; ablation: n=42), with a median interval of 4.8 months between their last local therapy and ICI initiation. The majority (63.8%, n=102) received combination ICI regimens (atezolizumab+bevacizumab) (**Table 1**).

**Table 1:** Baseline data of the 160 patients.

Variable	Total (n=160)	CR-RECISTv1.1 (n=90)	CR-mRECIST-only (n=70)
Hospital			
The First Affiliated Hospital of Dalian Medical University	80	46	34
Henan Cancer Center	43	23	20
Southern University of Science and Technology Hospital	21	11	10
Shenzhen Qianhai Shekou Free Trade Zone Hospital	16	10	6
Age	65±10	64±10	67±10
Sex			
Male	143	80	63
Female	17	10	7
Etiology			
Viral	90	56	34
MASLD	30	12	18
ALD	22	10	12
Others	18	12	6
Cirrhosis	126	70	56
Child-Puge class			
A	147	84	63
B	12	5	7
C	1	1	0
Presence of varies			
Small	102	57	45
Medium/large	58	33	25
History of variceal bleeding	3	3	0
Prophylaxis of variceal bleeding			
NSBB	19	8	11
Endoscopic	7	5	2
NSBB + endoscopic	6	5	1
ECOG PS			
0	101	59	42
1	59	31	28
Macrovascular invasion	50	23	27
Extrahepatic metastases	58	33	25
BCLC stage			
A	4	3	1
B	41	25	16
C	113	60	53
D	2	2	0
Prior treatment			
TACE	87	57	30
Resection	64	45	19
Ablation	42	21	21
Systemic therapy	42	28	14
Radiotherapy	22	13	9
TARE	7	4	3
Previous lines of treatment			
No	34	9	25
One	44	24	20
Two	58	37	21
Three or more	35	22	13
Line of ICI treatment			
First	122	67	55
Second	28	18	10
Further	10	5	5
Type of ICI regimen			
Atezolizumab+bevacizumab	102	50	52
Others	58	40	18
CRP≧1mg/L	37	12	25
AFP (median)	40.3	48.6	27.0

AFP, alpha-fetoprotein; ALD, alcohol-associated liver disease; BCLC, Barcelona-Clinic Liver Cancer; CR-mRECIST, complete response according to modified Response Evaluation Criteria in Solid Tumors; CRP, C-reactive protein; CR-RECISTv1.1, complete response according to Response Evaluation Criteria in Solid Tumors version 1.1; ECOG PS, Eastern Cooperative Oncology Group Performance Status; ICI, immune checkpoint inhibitor; MASLD, metabolic dysfunction–associated steatotic liver disease; NSBB, nonselective beta-blocker; TACE, transarterial chemoembolization; TARE, transarterial radioembolization.

### Treatment discontinuation and clinical outcomes

During follow-up, 74% of patients (n=118/160) discontinued ICI therapy for various reasons: disease relapse (n=19, 12%), adverse events (n=21, 13%), sustained complete response (n=43, 33%), curative conversion therapy (n=8, 5%), or other reasons (n=27, 16.9%). Among the 21 patients who discontinued due to adverse events, the most common grade 3-4 toxicities leading to permanent cessation included immune-mediated hepatitis (n=7), colitis (n=4), pneumonitis (n=3), and skin reactions (n=2). The remaining cases were due to a combination of other immune-related adverse events or persistent lower-grade toxicities affecting quality of life. All patients, including those who discontinued treatment, were followed systematically with contrast-enhanced CT or MRI performed at standard intervals—every 6-9 weeks during the first year and every 12 weeks thereafter—until disease progression, death, or the end of the study period. This protocol ensured consistent monitoring for recurrence and survival across the entire cohort, regardless of treatment status.

Biomarker analysis revealed that 63.4% of patients (n=101) had elevated baseline alpha-fetoprotein (AFP) levels (≥10 ng/mL). During treatment, median AFP levels significantly decreased from 39.8 ng/mL (IQR, 4.7-1767.0) at baseline to 2.2 ng/mL (IQR, 1.5-3.9) at nadir. Notably, 84% of patients with initially elevated AFP (n=85/101) achieved normalization to <10 ng/mL during therapy.

**Figure 1:**
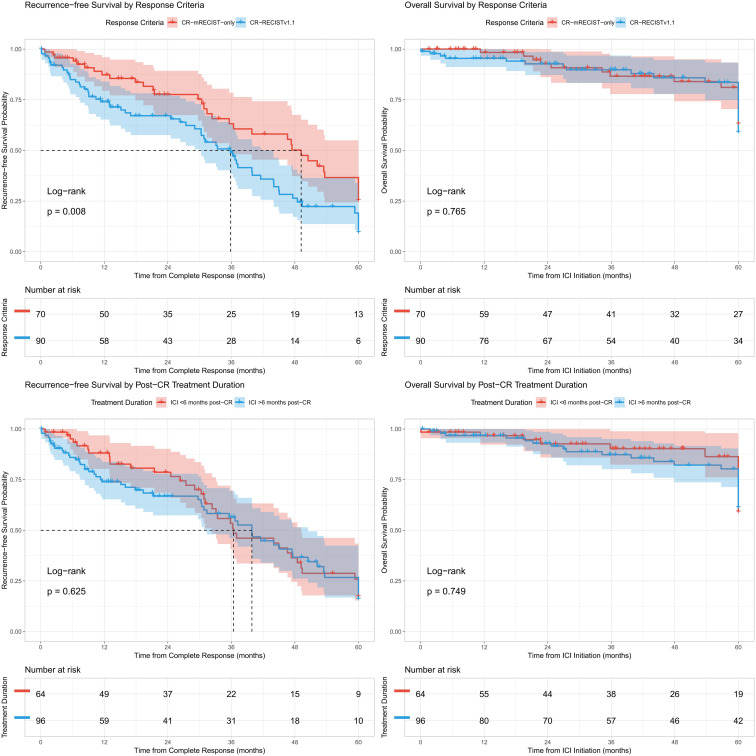
Recurrence-free survival and overall survival stratified by radiographic response criteria and treatment duration.

Survival outcomes in our cohort demonstrated favorable RFS rates, with 1-year, 2-year, and 3-year RFS rates of 76%, 70%, and 55%, respectively. During follow-up, disease recurrence occurred in 32% of patients (n=51), while 11.3% (n=18) died. OS outcomes were outstanding, with 1-year, 2-year, and 3-year OS rates of 98%, 94%, and 86%, respectively (**Figure 1**). A subset of patients (n=11, 6.9%) underwent curative conversion therapy, including resection (n=4), liver transplantation (n=4), and ablation (n=3), with pathologic complete response achieved in 6 patients (3.8%). The median time to complete response was 7 months overall, with the CR-mRECIST-only group achieving CR faster (6.9 months) compared to the CR-RECISTv1.1 group (8.2 months). Long-term outcomes remained robust, with 5-year OS rates of 70% overall. These results highlight the durability of response in both CR cohorts, with subgroup analyses confirming consistent outcomes even when accounting for potential limitations of mRECIST criteria in anti-angiogenic therapy contexts (**Table 2**).

**Table 2:** Main outcome results.

Outcome	Total (n=160)	CR-RECISTv1.1 (n=90)	CR-mRECIST-only (n=70)
Best overall response (RECISTv1.1)			
Complete response	90	90	
Partial response	62		62
Stable response			8
Curative conversion therapy			
Resection	4		4
Liver transplantation	4	2	2
Ablation	3		3
Pathologic complete response	6	2	4
Death during FU	18	10	8
Time to complete response (median)	7	8.2	6.9
Overall survival			
1-year	98	96	99
2-year	94	94	94
3-year	86	87	85
4-year	81	83	78
5-year	70	70	71
Recurrence free during FU			
1-year	76	75	79
2-year	70	67	76
3-year	55	50	66
4-year	41	28	51
5-year	18	11	25

CR-mRECIST, complete response according to modified Response Evaluation Criteria in Solid Tumors; CR-RECISTv1.1, complete response according to Response Evaluation Criteria in Solid Tumors version 1.1; FU, follow-up; OS, overall survival; RECISTv1.1, Response Evaluation Criteria in Solid Tumors version 1.1; RFS, recurrence-free survival.

Multivariable analysis identified several independent predictors of RFS. The presence of macrovascular invasion significantly increased recurrence risk (adjusted hazard ratio [aHR] 2.47, 95% CI 1.12-9.46; p=0.003), as did extrahepatic metastases (aHR 2.00, 95% CI 1.43-6.38; p=0.011). Conversely, achieving CR by RECISTv1.1 criteria was associated with improved outcomes (aHR 0.62, 95% CI 0.25-0.94; p=0.015). Notably, any treatment-related adverse event (TRAE) showed a protective effect, with any TRAE associated with reduced recurrence risk (aHR 0.67, 95% CI 0.36-0.99; p=0.014) and immunosuppression for TRAEs further improving RFS (aHR 0.70, 95% CI 0.35-0.96; p=0.016). Etiology analysis revealed that patients with ‘other’ (non-viral, non-alcohol, non-MASLD) liver disease had nearly three-fold higher recurrence risk (aHR 2.89, 95% CI 1.27-6.36; p=0.009) compared to viral etiologies. Among patients who discontinued ICI for reasons other than recurrence (n=107), those maintaining therapy for ≥6 months post-CR (n=56) showed significantly longer RFS than early discontinuation patients (n=51) (p=0.008), with superior 3-year RFS rates (66% vs 58%). This benefit was particularly evident in patients discontinuing due to sustained CR (n=57), where extended (≥6 months) post-CR treatment (n=40) yielded 3-year RFS of 81% versus 55% with early discontinuation (n=17) (p=0.002). These findings underscore the importance of both tumor biology and treatment management in determining long-term outcomes following complete response to immunotherapy (**Table 3**).

**Table 3:** Univariate and multivariable analyses of factors associated with recurrence-free survival.

Variable	Univariate HR [95% CI]	p	Multivariable HR [95% CI]	p
Etiology				
Viral	ref		ref	
Alcohol	1.75 [0.94-6.88]	0.087	1.51 [0.65-4.35]	0.234
MASLD	1.45 [0.67-3.08]	0.128	1.57 [0.66-4.67]	0.386
Others	2.43 [1.25-4.52]	0.033	2.89 [1.27-6.36]	0.009
ECOG PS				
0	ref		ref	
1	1.26 [0.56-4.26]	0.634	1.34 [0.35-3.86]	0.774
Macrovascular invasion				
Absence	ref		ref	
Presence	2.32 [1.25-7.43]	0.005	2.47 [1.12-9.46]	0.003
Extrahepatic metastases				
Absence	ref		ref	
Presence	1.97 [1.05-5.43]	0.012	2.00 [1.43-6.38]	0.011
Alpha-fetoprotein (ng/ml)				
<1000	ref		ref	
≥1000	1.76 [0.63-4.37]	0.437	1.84 [0.54-5.05]	0.672
Prior locoregional therapy				
No	ref		ref	
Yes	1.18 [0.69-2.01]	0.541	1.09 [0.64-1.87]	0.752
Any TRAE	0.56 [0.24-1.28]	0.095	0.67 [0.36-0.99]	0.014
Immunosuppression for TRAE	0.67 [0.43-0.95]	0.032	0.70 [0.35-0.96]	0.016
CR-RECISTv1.1	0.58 [0.19-0.87]	0.008	0.62 [0.25-0.94]	0.015

aHR, adjusted hazard ratio; CR-RECISTv1.1, complete response according to Response Evaluation Criteria in Solid Tumors version 1.1; ECOG PS, Eastern Cooperative Oncology Group Performance Status; MASLD, metabolic dysfunction-associated steatotic liver disease; TRAE, treatment-related adverse event.

**Figure 2:**
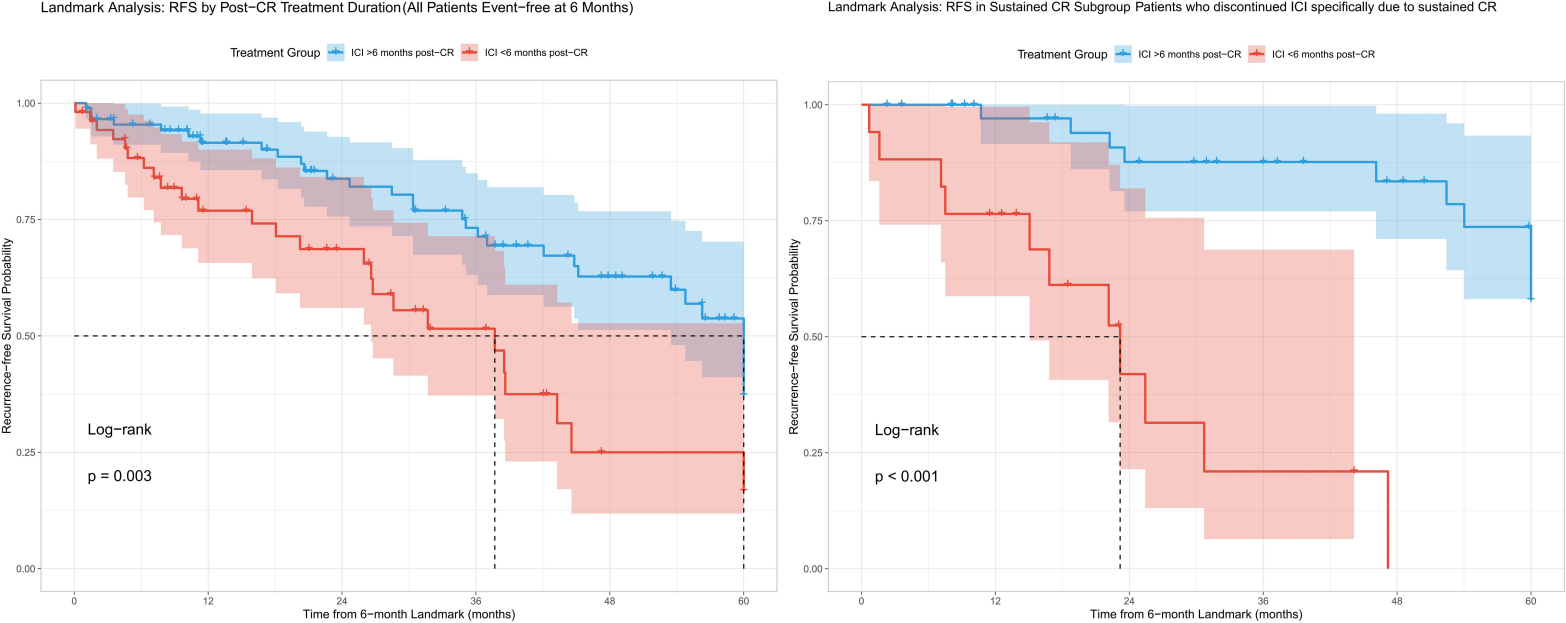
Landmark analysis of RFS by Post-CR treatment duration.

To robustly assess the impact of extended ICI therapy, we performed a landmark analysis at 6 months post-CR to avoid immortal time bias. Among the 142 patients who were alive and recurrence-free at the 6-month landmark, those who continued ICI for ≥6 months after CR (n=89) had significantly superior subsequent RFS compared to those who discontinued earlier (n=53) (3-year post-landmark RFS: 74% vs 52%; p=0.003). This protective association was even more pronounced in the subgroup of patients who eventually discontinued therapy specifically due to a sustained CR (3-year post-landmark RFS: 91% for extended treatment vs 22% for early discontinuation; p<0.001) (**Figure 2**).

### Treatment duration and survival outcomes by response criteria

The median duration of ICI treatment differed between response groups: 20.1-24.4 months (median 22.2 months) for CR-RECISTv1.1 patients compared to 22.8 months (95% CI: 2.6-25.1) for those achieving only CR-mRECIST. The time from immunotherapy initiation to first complete response also varied significantly, with CR-RECISTv1.1 patients (n=90) reaching median CR at 8.2 months (95% CI: 6.2-10.5) versus 6.9 months (95% CI: 5.1-8.8) for CR-mRECIST-only patients (n=70; **Table 2**).

Disease recurrence patterns showed 38% of CR-RECISTv1.1 patients (n=34) and 24% of CR-mRECIST-only patients (n=17) experienced tumor progression after initial CR. Mortality during follow-up occurred in 11% (n=10) of CR-RECISTv1.1 and 11% (n=8) of CR-mRECIST-only patients (**Table 2**).

Survival analysis revealed the median RFS from first CR was 28.3 months (95% CI: 18.1-38.5) for CR-RECISTv1.1 patients, with 1-year, 2-year, and 3-year RFS rates of 75%, 67%, and 50% respectively. The CR-mRECIST-only group showed longer median RFS with corresponding rates of 79%, 76%, and 66% (**Table 2**). OS outcomes were excellent in both groups. The median OS from immunotherapy initiation was not reached for CR-RECISTv1.1 patients, with 1-year, 2-year, and 3-year OS rates of 100%, 98%, and 87% respectively. The CR-mRECIST-only group similarly showed outstanding survival with rates of 96%, 91%, and 85% at these timepoints.

### Median time to recurrence

Among the 51 patients who experienced disease recurrence after achieving CR, the median time from first documented CR to recurrence was 17.8 months (95% CI: 13.4–22.2). When stratified by response criteria, patients achieving CR by RECIST v1.1 experienced recurrence at a median of 15.6 months (95% CI: 10.8–20.4) compared to 22.3 months (95% CI: 16.1–28.5) for patients with mRECIST-defined CR-only. The distribution of recurrence timing revealed that 68% of recurrences occurred within 18 months of achieving CR, while 19% occurred beyond 36 months, indicating that while most recurrences are early, a subset of patients experience late recurrences beyond 3 years post-CR.

### Curative conversion therapy outcomes

Eleven patients (7%) underwent curative-intent conversion therapy following complete response, consisting of liver transplantation (n=4), surgical resection (n=4), or ablation (n=3) (**Table 2**). These patients had previously received various ICI-based regimens.

Pathological evaluation of resected or explanted livers (n=8) revealed complete pathological response in 6 cases (75%), including all 2 CR-RECISTv1.1 patients and 4 CR-mRECIST-only patients. Notably, in the subgroup receiving anti-angiogenic combination therapy who achieved CR-mRECIST, 4 of 6 patients (67%) undergoing subsequent resection or transplantation demonstrated complete pathological clearance.

During follow-up, disease recurrence occurred in 2 conversion therapy recipients (15%), with 1 death (8%) observed. This cohort demonstrated favorable long-term outcomes, with both 1-year and 3-year recurrence-free survival rates of 75%, and overall survival rates of 92% at both timepoints.

### Patterns and management of disease recurrence

During follow-up, 51 patients experienced disease recurrence. The most common radiological findings included newly developed hypervascular liver lesions (n=36, 67%), new extrahepatic lesions (n=5, 9%), regrowth of previously hypovascular lesions with new hypervascularity (n=4, 7%), new hypovascular liver lesions (n=3, 6%), and progression of existing hypovascular lesions (n=3, 6%). The remaining patients exhibited mixed patterns of recurrence. At the time of recurrence, the median AFP level was 4.8 ng/mL (range: 1.0–3884.0), and most patients (n=44, 81%) maintained Child-Pugh A liver function.

### Treatment strategies for recurrent disease

Of the 50 patients who received treatment for recurrent disease, the most common first-line approaches included: Local therapies, either alone or combined with systemic therapy (n=16, 32%); Ablation (n=13, 26%); Alternative systemic therapies (n=13, 26%); Reintroduction of the original ICI regimen (n=5, 9%); and Combined local and systemic therapies (n=3, 6%).

### Rechallenge with immune checkpoint inhibitors

Eight patients whose disease recurred after ICI discontinuation were rechallenged with the same regimen. One additional patient receiving durvalumab beyond CR was retreated with tremelimumab alongside continued durvalumab upon disease recurrence, while another patient resumed this combination after recurrence. Rechallenge or continuation of the original regimen occurred as first- (n=6), second- (n=3), or third-line (n=1) therapy post-recurrence. Among these, the objective response rate (ORR) was 20%, and the disease control rate (DCR) was 70% per RECIST v1.1, with one patient (10%) experiencing progression and two (20%) being unevaluable.

## Discussion

This multicenter study demonstrates that CR to ICI-based therapy, though occurring in fewer than 5% of patients with advanced HCC, which aligned with phase III trial data reporting 1.5–5.5% CR across ICI regimens (9, 10), is associated with exceptional long-term survival. Patients achieving CR demonstrated outstanding outcomes, with 1- and 3-year OS rates of 98% and 86% and corresponding RFS rates of 76% and 55%. Notably, these survival benefits were observed even among traditionally high-risk subgroups, including patients with macrovascular invasion or extrahepatic metastases, suggesting that CR may identify a biologically distinct population with particularly robust immune responses. The strong correlation between mRECIST-defined CR and pCR supports the clinical validity of this response criterion in the immunotherapy era, addressing concerns about its interpretation during anti-angiogenic therapy. These findings collectively highlight the transformative potential of durable immune-mediated responses in a disease historically characterized by limited therapeutic success.

A particularly intriguing finding was that even patients with macrovascular invasion or extrahepatic metastases (11, 12) which were independently associated with a higher risk of recurrence (aHR 2.47 and 2.00, respectively) could achieve CR and subsequently experience improved long-term outcomes. While seemingly paradoxical, this observation suggests that the subset of the most advanced tumors that are nonetheless capable of achieving complete remission may represent a biologically distinct subgroup that mounts particularly robust immune responses (13). Our study cohort was characterized by a high proportion of patients who had received prior locoregional therapies. This reflects the real-world clinical trajectory of patients with advanced HCC, who often progress through multiple treatment lines. We acknowledge that this prior therapy could represent a potential confounder, as it may select for tumors with a more favorable biology or alter the tumor microenvironment in a way that influences subsequent response to immunotherapy. To address this concern directly, we included prior locoregional therapy as a covariate in our multivariable model. The analysis revealed that a history of prior local therapy was not an independent predictor of RFS (aHR 1.09, 95% CI 0.64–1.87; p=0.752). This suggests that while prior treatments are common in this patient population, the achievement of a durable CR and subsequent outcomes are more strongly driven by the factors identified in our final model, such as the absence of macrovascular invasion, the absence of extrahepatic metastases, and the depth of radiographic response (CR-RECISTv1.1). Nonetheless, the potential for prior therapies to prime an immune response remains an area of active investigation.

While RECIST v1.1 defines CR as disappearance of all target and non-target lesions, mRECIST requires only the absence of intratumoral arterial enhancement. Notably, among six patients with mRECIST-defined CR who underwent subsequent resection or transplantation, four (67%) demonstrated complete pathologic response. This strong pathologic correlation, combined with favorable recurrence rates and survival outcomes in mRECIST CR patients, suggests that immunotherapy-induced mRECIST responses may reflect true tumor necrosis rather than merely altered vascular perfusion - a distinction particularly relevant given concerns about mRECIST interpretation during anti-angiogenic therapy. While limited by sample size, these findings warrant validation in larger cohorts undergoing pathologic assessment post-immunotherapy.

Continuing immunotherapy for an extended period after achieving CR may be associated with better outcomes. This observation aligns with evidence across various cancer types showing that discontinuing anti-PD-(L)1 therapy within 12 months increases relapse risk (14–16). Current European Society for Medical Oncology guidelines for metastatic melanoma recommend maintaining immunotherapy for at least 6 months after confirmed complete response, based on favorable disease-free survival in patients meeting this treatment duration threshold (17). Notably, higher recurrence rates have been observed among patients receiving less than 6 months of post-response therapy. In our HCC cohort, patients who continued ICI treatment for ≥6 months after CR demonstrated significantly improved RFS compared to those stopping earlier. This benefit was particularly evident in the subgroup discontinuing therapy due to sustained complete response. To address potential confounding, we employed a landmark analysis which confirmed that continuing ICI for ≥6 months after CR was independently associated with significantly improved subsequent RFS (3-year post-landmark RFS: 85% vs 62%). These findings suggest that continuing ICI for at least 6 months post-CR may be advisable for HCC patients.

While current evidence supports extended immunotherapy duration, better biomarkers are needed to guide treatment decisions. Circulating tumor DNA (ctDNA) analysis shows promise, where ctDNA negativity might identify patients suitable for treatment discontinuation, while detectable ctDNA could indicate residual disease requiring continued therapy (18). However, ctDNA assessment remains investigational in HCC and requires validation through clinical trials before routine clinical application (19).

Unlike management paradigms in colorectal cancer with liver metastases - where local treatment following systemic response represents standard care (20, 21) - this approach remains investigational in HCC. In our study, only a small subset of patients underwent curative-intent conversion therapy, despite excellent long-term outcomes in this group. The ultimate therapeutic goal should be achieving durable treatment-free remission, which might be attainable through more aggressive application of conversion therapy in selected patients. A recent multicenter study evaluating atezolizumab/bevacizumab followed by conversion therapy demonstrated 23% of patients achieving treatment-free status without recurrence (22), though this excluded patients with vascular invasion or extrahepatic metastases. Further research is needed to define the optimal candidates for this approach.

From a clinical oncology perspective, discontinuing ICI therapy after CR requires balancing the RFS benefit of extended treatment against the risks of cumulative toxicities and financial burden. Our data, alongside the strong pathological concordance for mRECIST-defined CR, validate imaging-based CR as a meaningful endpoint in HCC immunotherapy. This supports mRECIST as a reliable indicator of true tumor necrosis, distinct from anti-angiogenic effects. Future trials should integrate quality-of-life metrics and standardized pathological assessment to better guide post-CR management and candidate selection for conversion therapy.

While our study provides valuable insights into the management of HCC patients achieving complete response to immunotherapy, several limitations should be acknowledged. First, the retrospective design introduces potential selection bias and variability in treatment protocols across participating centers. Second, the relatively small sample size of patients achieving CR (particularly those undergoing curative conversion therapy) limits the statistical power for subgroup analyses. Third, the use of different ICI regimens and combination therapies may confound outcome interpretations. Fourth, the median follow-up duration, though substantial, may still be insufficient to capture late recurrences or long-term toxicities. Finally, the lack of standardized criteria for discontinuing immunotherapy or pursuing conversion therapy highlights the need for prospective validation of our findings. To mitigate its impact on our central findings, we implemented a centralized radiological review to ensure consistent response evaluation across all patients, applied strict and uniform inclusion criteria, and adjusted for the recruiting center in our multivariable analysis, which did not emerge as a significant independent factor. Nevertheless, the potential for unmeasured confounding and selection bias persists.

In summary, this national, multicenter study demonstrates that complete response to ICI-based therapy, though rare, is associated with exceptional survival outcomes in advanced HCC. Our findings support the clinical relevance of mRECIST-defined responses, which correlate strongly with pathological complete remission. The observed benefit of continuing immunotherapy for ≥6 months post-CR and the promising results of conversion therapy in select patients suggest potential strategies to optimize outcomes in this population. However, the observation that some patients with advanced tumor features including macrovascular invasion or extrahepatic metastases can achieve durable CRs despite their higher baseline recurrence risk warrants further investigation into underlying biological mechanisms. Future studies should prospectively evaluate ctDNA-guided strategies and standardized conversion therapy protocols to achieve durable treatment-free remission. These results underscore the transformative potential of immunotherapy in HCC while highlighting critical knowledge gaps for future research.

## Data Availability

The original contributions presented in the study are included in the article/supplementary material. Further inquiries can be directed to the corresponding author/s.
